# Development of candidate HIV vaccines using VSV vectors to express membrane-anchored MPER immunogen

**DOI:** 10.1186/1742-4690-9-S2-P327

**Published:** 2012-09-13

**Authors:** M Yuan, A Wilson, M Kemelman, M Panis, I Lorenz, C Jurgens, MJ Chiuchiolo, R King, M Caulfield, CL Parks

**Affiliations:** 1 International AIDS Vaccine Initiative, Brooklyn, NY, USA

## Background

The HIV-neutralizing activity of monoclonal antibodies 2F5, 4E10, and Z13 have shown that the membrane-proximal external region (MPER) of HIV Env is an important vaccine target, but development of an immunogen that elicits similar virus-neutralizing antibody responses against the MPER from a wide range of subtypes has been difficult to achieve. It has been proposed that a MPER immunogen must be membrane-associated to adopt the conformation needed to elicit broadly neutralizing antibodies, and accordingly, we have developed Vesicular Stomatitis Virus(VSV) Vectors that express membrane-anchored MPER epitopes.

## Methods

Replication-competent and single-cycle VSV vectors have been developed that express a truncated VSV G protein (G-Stem) in which the natural G ectodomain is replaced with MPER sequence (GStemMPER). The nucleotide sequence of GStemMPER has been optimized by computer based algorithms to ensure genetic stability. Several VSV vector modifications are also introduced for directing immune responses towards MPER rather than G. Those strategies include relocation of GStemMPER and VSV G glycoprotein to achieve maximal MPER expression while minimize the anti-vector immunity, incorporation of different VSV G serotypes and pseudotyping single- cycle VSV vectors with various types of glycoproteins.

## Results

Western blot analysis confirmed expression of MPER epitopes. Assessment by FACS showed the cell surface MPER being recognized by 2F5 and 4E10 monoclonal antibodies. Immunoprecipitation study further proved incorporation of MPER into viral particles.

## Conclusion

Multiple VSV-GStemMPER vectors are being evaluated in small animal studies to compare their immunogenicity and whether the membrane-anchored form of the MPER immunogen elicits humoral responses with a broad range of HIV neutralizing activity.

